# Camellia Sinensis Mouthwashes in Oral Care: a Systematic Review

**DOI:** 10.30476/DENTJODS.2020.83204.1045

**Published:** 2020-12

**Authors:** Ali Tafazoli, Ehsan Tafazoli Moghadam

**Affiliations:** 1 Dept. Clinical Pharmacy, Faculty of Pharmacy, Shahid Beheshti University of Medical Sciences, Tehran, Iran; 2 Dept. of Orthodontics, School of Dentistry, Qazvin University of Medical Sciences, Qazvin, Iran

**Keywords:** Camellia sinensis, Dental care, Green tea, Mouthwashes, Mouthrinse, Oral hygiene, Tea

## Abstract

Herbal products are increasingly growing in the oral care market. Some of the related herbal compounds in this field have considerable clinical evidence for use in mouthwashes in their background. Camellia sinensis or tea plant has attracted numerous researchers of dentistry and pharmaceutical sciences, in recent years, for its biologic and medicinal properties. The effects such as anti-septic, anti-oxidative, and anti-inflammatory activities have made this plant a suitable candidate for preparation of mouthwashes. In this systematic review, we tried to find, evaluate, and categorize the sparse evidence in medical literature about Camellia sinensis mouthwashes. We explored three scientific databases with keywords including tea, dental care, Camellia sinensis, and mouthwashes and found 69 relevant studies including 41 randomized controlled trials (RCTs), which are generally proposing anti-microbial, anti-plaque, and analgesic indications for these tea formulations. Considering the main trend in clinical evidence and favorable safety profile, Camellia sinensis products are able to act as antiseptic, anti-plaque, and anti-inflammatory agents and can be used as useful mouthwashes in the future clinical studies and practice.

## Introduction

Herbal agents have been extensively entered into oral care products, in recent years. These compounds have interesting medical and physiochemical characteristics. Nowadays lots of manufacturers are using herbal ingredients in their products to provide additional therapeutic characteristics. Among these herbs, Camellia sinensis or tea plant has a unique phytochemical and pharmacological profile, which makes it a perfect candidate for use as active constituent in oral mouthwashes [ 1 ].

Numerous compounds have been discovered in tea plant with therapeutic effects. These beneficial properties are mostly connected to polyphenols, which usually account for 30% of the dry weight of the solid materials in the brewed green tea [ 2 ]. Anti-cancer effect of polyphenols of green tea has been evaluated in many in vitro studies [ 3 - 8 ]. An in vitro study on epigallocatechin gallate (EGCG), an active ingredient found in Camellia sinensis, showed that EGCG decreases the expression of phosphorylated epidermal growth factor receptors in oral cancer cells, as well as non-oral ones [ 7 ]. Cell cycle analysis on oral squamous cell carcinoma cells shows that EGCG induces G1 phase arrest and increases occurrence of apoptosis among tumor cells [ 9 ]. It has also been reported that green tea polyphenols possess anti-colorectal cancer activity, by altering intestinal microbiota and intestinal colonization of oral cavity bacteria that are associated with gastrointestinal malignancies [ 10 ]. Studies have also revealed anti-angiogenic effects for green tea catechins, which can suppress tumor growth [ 11 - 14 ]. Cardio-protective [ 15 - 16 ], cholesterol lowering [ 17 - 20 ], anti-hypertensive [ 21 - 23 ], and anti-diabetic [ 24 - 25 ] properties of polyphenols has also been indicated in many clinical studies and literature reviews. Neurological studies indicate that green tea polyphenols act as antioxidant compounds, which have neuro-protective benefits and modulatory function on intracellular signaling, and cell survival/death genes; hence, they can be useful in prevention and treatment of neurodegenerative diseases [ 26 - 30 ]. Antimicrobial, anti-inflammatory, and anti-nociceptive properties of Camellia sinensis extract have been the subject of many studies, as well [ 2 , 31 - 36 ]. Green tea antibacterial function has been related to its EGCG and other compounds through damaging the bacterial cell membrane [ 37 ], inhibitory effect against gyrase enzyme, and destroying cytoplasmic membrane of bacterium [ 38 ]. Anti-inflammatory function of EGCG is mainly exerted by down-regulation of Cyclooxygenase-2 via inhibition of interleukin-1b-dependent pro-inflammatory signal transduction and interleukin-6, interleukin-8 and tumor necrosis factor-α gene expression at inflammation sites [ 39 - 40 ]. These characteristics have attracted the scientists for substantial evaluations in local application of tea products. Accordingly, dental researches have also focused on tea-related formulations, in mouthwash forms, for treatment of several oral diseases [ 41 - 43 ].In this review, we tried to collect and categorize the potential applications of Camellia mouthwashes in different dentistry fields to demonstrate the most evidence-based indications with their advantages and disadvantages. 

### Search Strategy

In order to provide a systematic review with the highest accordance to *Preferred Reporting Items for Systematic Reviews and Meta-Analyses* (PRISMA) guidelines, besides a comprehensive and acceptably relevant data pool, we searched three highly accredited databases including PubMed, Cochrane library, and Google Scholar. We gave a priority to PubMed database, due to its privileges and scientific characteristics for systematic searching. 

### Inclusion criteria

The inclusion criteria involved journal articles, those with titles including “green tea” or “tea” or “Camellia sinensis” and “mouthwash” or “mouth rinse” or “gargle” in the field of dentistry. Our exclusion criteria were non-English-abstract articles and those focused on adverse effects or staining capabilities of green tea products. We explored PubMed database with MeSH terms, “tea”, and “mouthwashes” at the first step. We also tried “Camellia sinensis” and “mouthwashes” for MeSH terms, which yielded different results. Finally, using the search order ((*"Tea"[Majr]) OR "Camellia sinensis"[Majr]*) and *"Mouthwashes"[Majr]* provided our desired results to establish a basis for our literature review. Then we searched Google Scholar with the same keywords and arranged entries based on “relevance”. All the topics were screened, one by one, to the last and the suitable manuscripts were handpicked. Consequently, the Cochrane library was searched to look for potentially available reviews about this topic.

We limited the search results to English language articles. All the harvested articles were pooled together and the repeated ones were deleted. All the articles were meticulously evaluated, analyzed, and defragmented to categorize the sub-headings. We added several academic references from Google to develop our ideas, and state it more clearly.

**Figure 1:**
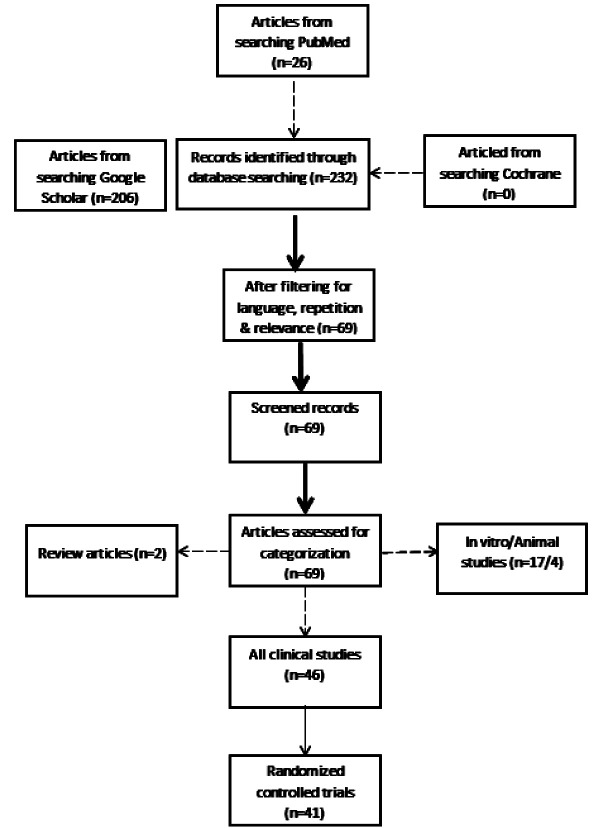
Flow diagram depicting the study selection and categorization process for “Camellia sinensis/tea” and “mouthwashes” search results

All the investigation was performed and reexamined with two experts in this field and the whole procedure was conducted according to guidelines of the ethical committee of Qazvin University of Medical Sciences. The systematic search flow-chart is depicted in Figure 1. 

### Literature Review Outcome

Our search protocol in PubMed yielded 26 journal articles. Among them, 25 were in English. Four papers were omitted due to their topic, which focused on green tea products as staining agents, without assessment of potential therapeutic effects. Another article, which was unintentionally included due to the search engine intrinsic malfunction, was also considered irrelevant and omitted. There were two in vitro studies, one review, two clinical trials without randomization and 15 RCTs. Searching Google Scholar added 15 in vitro studies, one review, four animal studies, three non-randomized trials and 26 RCTs to the reference list. The similar search process in the Cochrane database did not yield any articles.

It could be stated that almost every clinical study, evaluated a different specification of the mouthwash from a different point of view. However, those with common features were gathered together in order to present a well-organized categorization for topics. The number of articles was limited and our goal was only gathering and categorization of the evidence. Therefore, performing a meta-analysis was relinquished, intentionally. 

## Discussion

In this part, we classified the beneficial effects of different tea-based local oral formulations based on the type of scientific evidence and potential clinical indications. These involve both basic experimental studies with clinical implications and clinical trials.

### *In vitro* and animal studies

Anti-bacterial properties were the major focus of numerous pre-clinical studies on tea products. In a study for evaluation of antibacterial efficacy of an extemporaneous green tea-containing mouth rinse on oral pathogens including *Streptococcus mutans* (S. mutans), *Streptococcus sanguis, Enterococcus faecalis, Pseudomonas aerogenosa* and *Escherichia coli*, the herbal formulation showed considerable inhibitory effects on all microorganisms, although weaker than chlorhexidine (CHX) [ 44 ]. A study evaluating antibacterial effect of green tea extract on multi-drug resistant pathogens showed inhibitory effect on *Escherichia coli* and *Streptococcus aureus* and strong synergistic suppression against pathogens in combination of EGCG with gentamycin. This in vitro observation suggested that green tea has stronger inhibitory effect on gram-positive pathogens than gram-negative ones [ 45 ]. A study was conducted in Laval University, Quebec, Canada on inhibitory effect of green tea extract on virulence markers of *Fusobacterium nucleatum*, a key factor in initiation and progression of periodontal diseases. The results indicated that treatment of pre-formed *Fusobacterium nucleatum* biofilm with green tea extract caused a time-dependent decrease in biofilm viability and decreased adherence of *Fusobacterium nucleatum* to oral epithelial cells [ 46 ].

Anti-fungal property of green tea has been the subject of many in vitro studies, as well. Sitheeque *et al*. [ 47 ] showed *Candida glabrata* is the most sensitive species of Candida to tea polyphenols followed by *Candida parapsilosis* and *Candida albicans* (C. albicans). Since being the most common fungal species found in oral cavity, anti-fungal activity of green tea is mostly evaluated against C. albicans [ 48 ]. It is suggested that the EGCG found in green tea acts as an antifolate compound, which disturbs metabolism of folic acid in C. albicans [ 49 ]. Another study showed that polyphenolic compounds found in green tea extract, caused 75% reduction in viable C. albicans cells during biofilm formation. This research proposed that polyphenols anti-fungal activity is exerted via reduction of proteasomal activity in C.albicans cells [ 50 ]. Another study conducted by Yadegari *et al*. [ 51 ], demonstrated that inhibitory effect of EGCG against C. albicans is time dependent. The results also showed that EGCG could be effective on fluconazole-resistant C. albicans. It has also been stated that green tea aqueous extract can be useful against C. albicans colonies growing on acrylic resin and polyvinyl chloride surfaces used in prosthetic dentures and orthodontic appliances [ 52 - 53 ]. In a study conducted by Mollashahi *et al*. [ 54 ], on tooth substrate of extracted premolar teeth, it was implied that green tea extract has the potential to be used as an irrigating agent in endodontic treatments due to its anti-fungal properties. However, the researchers of the study suggested evaluating its biocompatibility and safety before using it as an irrigating solution in clinical settings. Another study in the department of biotechnology in Graphic Era University, India, investigated synergistic activity of green tea extract and fluconazole, amphotericin B and copper sulphate as a combinational therapy against C. albicans. The results proved a synergic effect that can be efficient against C. albicans and implied that the combinational therapy does not show cytotoxic activity on healthy cells [ 55 ].

Some in vivo experiments have studied green tea’s anti-inflammatory function. An animal study on type 1 diabetic rats showed green tea extract minimizes expression of receptor activator of nuclear factor kappa-Β ligand and tumor necrosis factor-alpha, consequently decelerating alveolar bone resorption compared to placebo group [ 56 ]. Another animal observation on albino rats using green tea extract, illustrated a significant reduction in nicotine-induced damage, in terms of inflammatory cell infiltrates in buccal mucosa [ 57 ]. Such observations proposed the potential benefits of tea mouthwash preparations in clinical application. However, there are some controversial findings. Regarding the wound healing, an animal study failed to show a significant effect for green tea formulation [ 58 ].

### Clinical trials and RCTs

Screening and assessment of clinical trials including RCTs in this review revealed a growing trend for utilization of Camellia products in dentistry clinical practice. Almost every group of authors examined a new and divergent application for such formulations, which are categorized in the following sections. Specifications of the RCTs are summarized in Table 1.

**Table 1:** Randomized controlled trials of camellia sinensis mouthwashes

Author/Year	Indication	Product	Number of Participants	Finding	Adversities	Reference
Hajiahmadi/2019	Antiseptic (for oral cavity)	Green tea leaves extract mouthwash 5% / Green tea-Xylitol 20% mouthwash	64	Higher antibacterial efficacy in green tea- Xylitol than green tea mouthwash	No adverse effects reported	[97]
Salama/2019	Antiseptic (for oral cavity)	Green tea leaves extract mouthwash	40	Decreased S.mutans colony count compared to control group	NA	[65]
Yaghini/2019	Plaque removal	Green tea- Loe vera extract mouthwash	60	Decreased PI and GI compared to CHX and martica mouthwash	No adverse effects reported	[99]
Khanchemehr/2019	Oral health maintenance	Green tea leaves extract mouthwash 5%	46	Oral health setting improvement compared to CHX	No adverse effect reported	[70]
Mustafa/2019	periodontal parameters and inflammatory markers improvement	Green tea leaves extract mouthwash	45	improvement in clinical periodontal parameters and inflammatory markers compared to CHX	NA	[91]
Ahmadi/2019	Antiseptic (for oral cavity)	Green tea leaves extract mouthwash %	30	Decreased microbial count compared to green tea gel 0.5%	NA	[75]
Romoozi/2018	Plaque removal	Green tea leaves extract mouthwash	10	Decreased PI and GI but less than CHX group	No adverse effect reported	[78]
Nagar/2018	Plaque removal	Green tea leaves extract mouthwash 1 mg/dl	30	Decreased PI compared to CHX and white tea extract	No adverse effect reported	[83]
Khanchemehr/2018	Antiseptic (for orally intubated patients)	Green tea leaves extract mouthwash 5%	46	Decreased pharyngeal microbial load compared to CHX	NA	[71]
Prabakar/2018	Antiseptic (for oral cavity)	Green tea leaves extract mouthwash	52	Less effective than CHX in decreasing S.mutans colony count	NA	[61]
Elvina/2018	Analgesic (for orthodontic treatments)	Green tea leaves extract mouthwash	40	Less effective compared to Paracetamol	NA	[95]
Ghorbani/2018	Denture stomatitis treatment	Green tea leaves extract mouthwash 0/5%	22	Decreased size of denture stomatitis lesion compared to Nystatin 100000 u/ml	No adverse effect reported	[69]
Shalini/2018	Plaque removal	Green tea leaves extract mouthwash	32	Decreased PI less than CHX and herbal mouthwash (HiOra)	No adverse effect reported	[88]
Kamalaksharappa/2018	pH modulation	Green tea leaves extract mouthwash 2%	40	Increased salivary pH compared to probiotic	NA	[86]
Thomas/2017	Antiseptic (for oral cavity)	Green tea leaves extract mouthwash 0/5%	45	Decreased microbial count compared to garlic-lime and NaF 0.05%	No adverse effects reported	[63]
Hegde/2017	Antiseptic (for oral cavity)	Green tea leaves extract mouthwash 0/5%	75	Decreased S.mutans and lactobacilli spp. colony count but less than CHX group	No adverse effects reported	[68]
Goyal/2017	Antiseptic (for oral cavity)	Green tea leaves extract mouthwash	30	Decreased S.mutans colony count compared to saliva	No adverse effects reported	[66]
Raju/2017	Plaque removal in orthodontic patients	Green tea leaves extract mouthwash	30	More effective in decreasing plaque score than CHX and Listerine	No adverse effects reported	[87]
Ide/2017	Antibiotic prophylaxis	Bottled green tea solution with catechins concentration of 37 mg/dL	747	Minimal positive effect on flu incidence	No adverse effects reported	[74]
Abdulbaqi/2016	Plaque removal	Green tea- Salvadora persica extract mouthwash 0.25mg-7.82mg/ml	14	Decreased PI compared to CHX	No adverse effects reported	[96]
Thomas/2016	Antiseptic (for oral cavity)	Green tea leaves extract mouthwash 0/5%	30	Decreased S.mutans and lactobacilli spp. colony count compared to CHX	No adverse effects reported	[62]
Nandan/2016	Antiseptic (for oral cavity)	Green tea leaves extract mouthwash	60	Decreased S.mutans colony count compared to CHX	NA	[67]
Tafazoli Moghadam/2016	Analgesia (after periodontal surgery)	Green tea- Aloe vera mouthwash	45	Significant analgesic effect compared to placebo	No adverse effects reported	[43]
Sargolzaei/2016	Plaque removal	Green tea- Aloe vera mouthwash	60	Decreased PI compared to CHX	NA	[98]
Sarin/2015	Plaque removal	Green tea leaves extract mouthwash 2%	110	Decreased PI and GI compared to placebo	No adverse effects reported	[77]
Lamba/2015	Anti- inflammation	commercially available pre-formulated Colgate Plax Green Tea mouthwash	60	Decreased total leukocyte count and PI compared to saline mouth rinse	No adverse effects reported	[92]
Biswas/2015	Plaque removal	Green tea leaves extract mouthwash	48	Decreased PI compared to CHX	NA	[82]
Hambire/2015	Plaque removal	Green tea leaves extract mouthwash 0/5%	60	Decreased PI and GI compared to CHX and NaF 0.05%	No adverse effects reported	[85]
Shaban/2015	Alveolar osteitis prevention after surgery	Green tea leaves extract mouthwash 5%	57	No effect on alveolar osteitis compared to placebo mouthwash	No adverse effects reported	[72]
Radafshar/2015	Plaque removal	Green tea leaves extract mouthwash with 1% tannin	40	Decreased PI and GI compared to CHX	tooth staining because of tannins	[79]
Priya/2015	Plaque removal	Green tea leaves extract mouthwash 5%	30	Decreased PI and GI compared to CHX	No adverse effects reported	[80]
Neturi/2014	Antiseptic (for oral cavity)	Green tea leaves extract mouthwash 2%	30	Decreased microbial count compared to CHX	No adverse effects reported	[64]
Shahakbari/2014	Analgesia (pericoronitis)	Green tea leaves extract mouthwash 5%	97	Decreased pain score and painkiller use compared to CHX	NA	[94]
Ide/2014	Antibiotic prophylaxis	Bottled green tea solution with catechins concentration of 37mg/dL	747	No effect on flu incidence compared to tap water	No adverse effects reported	[73]
Eshghpour/2013	Analgesia (post-surgical)	Green tea leaves extract mouthwash 5%	43	Decreased pain score and painkiller use compared to placebo	No adverse effects reported	[93]
Balappanavar/2013	Plaque removal	Green tea solution mouthwash 0.5%	30	Decreased PI and GI compared to CHX	No adverse effects reported	[76]
Rassameemasmaung/ 2012	halitosis	Green tea solution mouthwash	60	Decreased VSCs level compared to placebo mouthwash	No adverse effects reported	[104]
Forouzanfar/2012	Plaque removal	green tea aqueous extract can	34	Decreased PI , GI and BOP compared to placebo	No adverse effects reported	[81]
Faria/2011	Antiseptic (for suture materials)	Green tea leaves water-ethanol extract mouthwash 25%	18	Decreased microbial count compared to CHX	NA	[60]
Esimone/2001	Antiseptic (for oral cavity)	Tea leaves extract 8% of two commercially available brands	30	Decreased microbial count with longer effect compared to Minty Brett	NA	[59]
Attin/1995	Plaque removal	Darjeeling first flush tea infusion	30	No difference from tap water	NA	[84]

Abbreviations: CHX: Chlorhexidine; GI: Gingival Index; NA: Not Available; PI: Plaque Index

### Randomized controlled trials

At first, we explored the real-life indications of green tea mouthwashes in RCTs in order to provide a more accurate insight toward clinical applications of these products.

### Antiseptic mouthwashes

As mentioned before, microbial regulation is the main purpose of using tea rinses in multiple studies. In a clinical trial on 30 healthy volunteers, gargling with two commercial tea extract solutions for 60 seconds resulted in significant decrease in microbial counts of expectorated oral rinses. Compared to a synthetic antiseptic mouthwash, those herbal oral rinses showed prolonged activity [ 59 ]. In addition to teeth and oral cavity, the effect of mouthwashes on devices and surgical derbies has also been assessed. In another RCT, performed on 18 patients with unerupted maxillary third molars with extraction indication, researchers tried to evaluate the anti-microbial and anti-adherence activity of different mouthwashes on suture materials after the surgery. Participants were equally divided in three groups of Camellia sinensis, *Calendula officinalis*, or CHX mouthwash users for one week. Compared to no intervention, as a control, all solutions were effective to reduce the number of adhered microorganisms, but the only significant difference was observed for CHX [ 60 ]. The same results was found in an RCT done by Prabakar *et al*. [ 61 ], in which they showed that CHX and green tea mouthwashes are significantly effective against S.mutans, although this antibacterial effect was superior for CHX, again. Another RCT compared the efficacy of green tea and CHX mouthwashes against S. mutans, Lactobacilli spp., and C. albicans in children with severe early childhood caries. Thirty children aged 4-6 years were randomly divided into green tea and CHX groups and were asked to use the prescribed mouthwashes once daily, for two weeks. The results showed significant decrease in S. mutans and Lactobacilli spp. colonies but no decrease was detected in C. albicans in both groups. This study revealed that green tea mouthwash was more efficient against S. mutans, whilst CHX had higher efficiency against Lactobacilli spp. Regarding tolerability and acceptability, this research proved green tea mouthwash, to be tolerable than CHX in children [ 62 ]. The same results for efficacy of green tea against S. mutans were reported in five other similar RCTs [ 63 - 67 ]. However, this privilege was not a fixed rule in all clinical trials [ 68 ]. Another clinical study showed green tea mouthwash could be as effective as nystatin in reducing candidal denture stomatitis symptoms [ 69 ].

In 2019, a crossover clinical trial was conducted on 46 patients admitted in the intensive care unit of Kerman hospital, Iran, using green tea and CHX mouthwashes for the test subjects on different days with a randomized order. No significant differences in oral health settings were observed between those mouthwashes at the end of the study [ 70 ]. In another experiment, the same treatment protocol was implemented on intubated patients admitted to intensive care unit for evaluation of bacterial colonies in their throat culture. Results of this study showed green tea and CHX had the same efficacy in reducing pharyngeal bacterial load [ 71 ]. A crossover RCT in Mashhad University of Medical Sciences evaluated incidence of alveolar osteitis after using green tea mouthwash following third molar surgery. The intervention resulted in no clinically significant difference for the mouthwash users compared to control group [ 72 ].

Besides local cleansing effects, green tea gargle has also been evaluated for prevention of airborne systemic infections. Ide *et al*. [ 73 ] randomized 757 high school children into green tea and tap water gargle three times daily during the influenza epidemic season. Despite the fact that no significant advantage was observed for green tea for flu prevention, authors stated that modification of the non-blinded design, controlling for better adherence rates in participants, and utilization of better placebos or diagnostic tools would probably reveal more promising results in the future studies. In a secondary analysis of these results using Bayesian estimation, the authors found that green tea gargling has a slight superiority over water gargling for that indication [ 74 ]. 

A clinical comparative evaluation between green tea mouthwash and green tea gel showed no significant difference in their antibacterial potency [ 75 ]. Considering CHX as an old and well-known oral disinfectant, tea mouthwashes have shown relatively acceptable anti-microbial effects without any of the complications associated with synthetic chemicals, including the adverse effects.

### Anti-plaque mouthwashes

Dental hygiene has also been evaluated in tea mouthwash trials. Potential anti-plaque effects have been investigated following the observations for effective anti-microbial properties of these compounds. A randomized blinded controlled trial compared anti-plaque potency and gingival protective effects of tea mouthwash solution with CHX and neem extract mouthwashes. By assigning 30 healthy participants to three groups, results showed that use of tea mouthwash was more effective in terms of plaque-score. In addition, it improved gingival and oral hygiene status. There was no placebo group in this trial [ 76 ]. In a larger RCT on 110 industrial male workers with mean plaque, index (PI) of 1.5 and gingival index (GI) of 1, participants were divided into green tea extract and placebo mouthwash groups. They were instructed to use the rinses twice daily, 30 seconds each time for 28 days. Results revealed that the PI and GI were significantly decreased in the green tea group [ 77 ]. Another comparative RCT conducted by Romoozi *et al*. [ 78 ], demonstrated that besides being as effective as CHX mouthwash in improving PI and GI in patients with plaque-induced gingivitis, tea mouthwash has the privilege of no dental staining which is a common side effect of CHX. Moreover, four RCTs with similar protocols have also verified the effectiveness of green tea mouthwash at the levels of CHX in improving plaque and gingival indices in patients with plaque-induced gingivitis [ 79 - 82 ]. On the other hand, in an RCT conducted in 2018 by Nagar *et al*. [ 83 ], 30 dental students whom were divided into CHX, green tea, and white tea groups were asked to use the prescribed mouthwashes for 10 days. According to the results, in spite of significant improvements in gingival and plaque indices by herbal formulations, they were less effective compared to CHX. In addition, in a single-blind, one-operator, three-period, three-treatment clinical study with a randomized Latin square cross-over design on 30 volunteers, no significant reduction in plaque surface area was detected for tea mouthwash compared to a synthetic commercial product [ 84 ].

Regarding the saliva acidity, an RCT showed green tea mouthwash to be more effective in increasing salivary pH than CHX and sodium fluoride mouthwashes [ 85 ]. In comparison with probiotics, green tea mouth rinse demonstrated similar success in increasing salivary pH, which can be beneficial in prevention of enamel demineralization [ 86 ].

Plaque control and oral hygiene maintenance has always been a major concern in orthodontic patients. In an RCT on 30 orthodontic patients with fixed orthodontic appliances, anti-plaque efficacy of CHX (as a routine prescribed mouth rinse in orthodontic treatments) and green tea mouthwash was compared. This study showed superiority in plaque reduction for green tea over CHX and Listerine mouthwash [ 87 ]. Contrarily, in another RCT, during fixed orthodontic treatment green tea exerted significant plaque reduction, while it was not as efficient as CHX [ 88 ]. 

C-reactive protein and alkaline phosphatase are considered two nonspecific inflammatory markers, which rise in peripheral blood circulation and gingival crevicular fluid as a sign of active periodontitis [ 89 - 90 ]. An RCT on 45 patients with mild to moderate localized chronic periodontitis showed that administration of green tea 5% mouthwash for two weeks significantly reduced these biomarkers in blood circulation in chronic periodontitis [ 91 ]. Lamba *et al*. [ 92 ] have also proposed that using green tea mouthwash after the phase I of periodontal treatments (Oral hygiene education, scaling, and root planning) had significantly higher effect in reducing the total leukocyte count, especially neutrophil count, compared to placebo. 

Anti-plaque effect of tea rinses is mainly related to its anti-microbial effect but presence of other mechanisms cannot be ruled out. It is almost a proven fact that these products can be efficiently used for plaque fighting. 

### Painkiller mouthwashes

Oral pain is a very common manifestation of numerous clinical presentations and pathologies in dental patients. Oral lesions and surgical wounds are on the top rank etiologies. Healing properties have been detected for tea ingredients. Accordingly, some studies evaluated relieving effects of such rinses. In a clinical trial with split-mouth double blind design, on 44 candidates for impacted third molar removal, the efficacy of green tea mouthwash was evaluated for post-operative pain management. Findings showed that daily application of this mouthwash significantly decreased the pain severity and number of required painkiller pills during the first 7 days after the surgery. No considerable adverse effect reported by herbal mouthwash users [ 93 ]. In addition, the analgesic effect of green tea has been evaluated on pericoronitis pain. Randomization of 97 patients with acute pericoronitis of mandibular third molar to green tea and CHX mouthwashes, revealed a significant decrease in pain score and number of painkiller uses and a non-significant improvement in trismus with herbal rinse [ 94 ]. Contrarily, in a clinical trial on orthodontic patients, green tea mouthwash was not as capable as acetaminophen in pain reduction during orthodontic tooth movements [ 95 ].

Generally, it could be stated that tea preparations are moderate painkillers. They have acceptable anti-nociceptive effects. These characteristics are sometimes inferior compared to common painkillers but bypassing numerous adverse effects is a considerable advantage.

### Combination mouthwashes

Several RCTs assessed the synergistic efficacy of Camellia products in combination with other herbal or chemical ingredients in mouthwash formulations. These studies provide the chance for designing formulations with higher effectiveness associated with acceptable safety. Based on in vitro findings about synergistic anti-bacterial and anti-adherence effects of green tea and Salvadora persica extracts on plaque and gingivitis producing bacteria, in a double-blinded, randomized crossover trial with 14 participants, efficacy of this combination was evaluated, clinically. Comparison of the test mouthwash with placebo and CHX mouthwashes showed that lowest plaque score was yielded by herbal product followed by CHX and then the placebo was ranked, with statistically significant differences [ 96 ]. In another clinical study, combination of tea extract with sodium lauryl sulfate in a mouthwash produced a greater anti-bacterial potency than the extract alone [ 59 ]. A randomized clinical evaluation between “green tea” and “green tea with Xylitol” mouthwashes, regarding their efficacy against salivary S. mutans and Lactobacillus colonies, findings demonstrated that addition of Xylitol to green tea extract had better antibacterial efficacy compared to green tea extract alone [ 97 ]. Some studies have indicated that combining green tea and Aloe vera extracts creates a more potent antimicrobial herbal mouthwash. An RCT on patients suffering from periodontitis showed combination of Camellia sinensis and Aloe vera extract has been highly effective in improvement of gingival indices [ 98 ]. Another comparative clinical trial between Aloe Vera-Green Tea, CHX, and matrica mouthwashes displayed that Aloe Vera-Green Tea mouthwash was as effective as CHX in reducing PI, GI, and bleeding on probing index and exerts more anti-plaque potency than matrica mouthwash [ 99 ].

### Other clinical trials

There are some clinical trials in scientific literature about Camellia mouthwashes, which do not have the straightforward RCT design. We evaluated these studies separately in this part. A clinical study conducted in 2019 tried to evaluate green tea mouthwash anti-bacterial efficiency by comparing organic acids found in saliva of 15 healthy adults before and 4 weeks after using the mouthwash. Researchers announced that green tea mouthwash has the potential to reduce bacterial activities in oral cavity via reduction of lactate concentration in saliva [ 100 ]. In a highly cited article by Ooshima *et al*. [ 101 ], the anti-plaque disposition effect of oolong tea was evaluated in 35 volunteers. Participants were instructed to rinse their mouth with tea extract ethanolic solution before, after each meal, and at bedtime. The findings were recorded and controlled with the use of an ethanol-only solution after one week. Results showed a significant inhibition of plaque disposition with the herbal tea solution.

In a quasi-experimental study, 81 orally intubated patients hospitalized in intensive care unit of a medical center in Taiwan were divided into three oral care protocol groups: green tea, boiled water, and control group. This study revealed irrigation with green tea extract in orally intubated patients could minimize mucosal changes and dental plaque accumulation and would be beneficial in improving their oral health status [ 102 ].

The efficacy of green tea mouthwash in management of halitosis has also been evaluated. In a clinical trial (without any statement about randomization) on 30 volunteers free of natural halitosis, participants were tested with four different mouthwashes including *Camellia sinensis* and *Curcuma zedoaria* aqueous solutions, CHX and water as a placebo, concomitant with episodes of cysteine challenge tests. All mouthwashes demonstrated acute inhibitory effects on volatile sulphur compounds but a lasting effect was only presented after use of CHX [ 103 ]. Another RCT, concerning halitosis, showed that green tea mouthwash could reduce 36.8 % of volatile sulphur compounds in patients who presented more than 80 parts per billion of these compounds in their morning breath [ 104 ]. 

## Conclusion

Camellia sinensis mouthwashes have shown acceptable efficacy for management of various oral pathologies. To our knowledge, this is the first review in medical literature, which involves all potential applications of green tea formulations in the form oral rinses, as an industrially standard medication in the setting of dentistry. Although there are several review articles about use of green tea in dentistry fields, none of them has the same comprehensiveness. Regarding our investigations, advantages of this herbal product has been demonstrated in the majority of studies. Therefore, some indications can be proposed for the tea mouthwashes with higher level of evidence including oral disinfection, dental plaque removal, and oral analgesia. An important and interesting issue in this context is the noticeable safety profile of these formulations, which is an outstanding advantage for this herbal mouthwash over synthetic chemical ones.
